# Slushies before school: exploring how nearby food outlets impact the eating behaviours of secondary students in Perth, Western Australia

**DOI:** 10.1186/s12889-026-26933-0

**Published:** 2026-03-17

**Authors:** Sharonna Mossenson, Frith Klug, Joelie Mandzufas, Jacinta Francis, Siobhan Hickling, Lukar Thornton, Gina Trapp

**Affiliations:** 1 Nutrition and Health Innovation Research Institute, School of Medical and Health Sciences, Edith Cowan University, 270 Joondalup Drive, Joondalup, WA Australia; 2 The University of Western Australia, Perth, WA Australia; 3 The Kids Research Institute Australia, Perth, WA Australia; 4 Cardiovascular Epidemiology Research Centre, School of Population and Global Health, The University of Western Australia, Perth, WA Australia; 5 Department of Marketing, University of Antwerp, Antwerp, Belgium

**Keywords:** School food environment, Adolescents, Dietary behaviour, Nutrition policy

## Abstract

The external school food environment, comprising food outlets and marketing near schools, shapes what young people eat. Yet little is known about how Australian adolescents perceive and navigate these environments. Focus groups were conducted with 31 school students (Years 7–12) from Perth, Western Australia, to explore their experiences of the external school food environment and its influence on their food-related behaviours. Transcripts were thematically analysed and six themes were identified. *Accessibility* emphasised proximity, transport links, opening hours and speed of service, with older students using outlets as social spaces. *Availability* of slushies, energy drinks, snacks and fast-food meal deals influenced purchasing, while school nutrition policies offered limited deterrence. *Affordability* was central, with supermarkets and convenience stores perceived as better value than the school canteen. *Advertising* through signage, buses and social media stimulated cravings and reinforced outlet visibility. *Acceptability and attitudes* reflected the social desirability of fast-food outlets, peer pressure, stigma around healthy eating and anti-social behaviours. Students proposed *strategies to improve the healthfulness of the external food environment*, including placing restrictions on unhealthy food outlets near schools, healthier alternatives, subsidies for nutritious foods and health promotion campaigns. Overall, this study found that adolescents perceive their external school food environment as cheap, convenient and socially compelling, but overwhelmingly unhealthy. These environments reinforce poor dietary habits and normalise harmful behaviours. Urgent, co-ordinated policy action is required. Limiting unhealthy outlet density near schools and reshaping pricing and promotion strategies are essential measures to protect young people from a food system that exploits their vulnerability.

## Introduction

Obesity among adolescents is a major public health concern, with prevalence increasing globally. In Australia it is estimated that 8% of adolescents are living with obesity [1, 2]. Obesity during adolescence often persists into adulthood and is linked to a higher risk and earlier onset of numerous chronic diseases [3, 4]. While obesity arises from a complex interplay of genetic, behavioural, socio-cultural factors [3, 4], the food environment has emerged as a significant driver [5]. The food environment encompasses the spaces where people interact with the food system; acquiring, preparing, and consuming food within specific physical, economic, and policy contexts [6]. For adolescents, these environments can include the home, school and community settings, such as grocery stores, supermarkets, restaurants and other food outlets [7].

Given that adolescents spend more time in and around school than in almost any other setting [8, 9], schools are a particularly influential food environment. The *internal school food environment* refers to food and beverages available within school grounds [10], (e.g., canteens (or ‘tuck shops’ / ‘cafeterias’) and vending machines). In contrast, the *external school food environment* encompasses the food outlets accessible to students on the journey to or from school [11]. In some schools, open gate policies allow students to leave the grounds during lunch breaks. Outdoor food advertising is also considered part of the external school food environment, providing frequent exposure to promotional material [12] that can influence students’ food preferences and purchasing behaviour [13].

External school food environments, defined by the food retail outlets accessible to students in the vicinity of schools, are often characterised by a high density of fast-food outlets and other retailers offering unhealthy food [14]. Evidence suggests that the concentration of these outlets has grown across developed countries, disproportionately clustering *around schools* in disadvantaged areas and thereby compounding existing health inequities and further entrenching obesity-related disparities [9, 14–17]. The school food environment is increasingly recognised as a setting where structural factors such as outlet density, food pricing and advertising, shape adolescent diet and obesity risk [5, 14].

Quantitative studies examining the relationship between obesity and the density of food outlets near schools have reported mixed findings, with some identifying positive associations and others finding little or no effect [18]. These inconsistencies may be explained by methodological weaknesses including variability in study design, exposure definitions, buffer distances and outcome measures [18]. Given these limitations [7], qualitative research offers an important complementary approach, allowing deeper exploration of how adolescents perceive and engage with the external food environment [19–22].

Adolescents are active social agents [23] and have “*a lot to say about why they eat what they eat*” [7, p. 186]. A recent narrative review echoes this sentiment and emphasises that young people should be considered as active participants within their food environments and that their eating behaviours are shaped by multiple influencing factors [24]. Indeed, international qualitative studies have shown that adolescents’ food choices are influenced by cost, convenience, availability and social norms. For example, Kelly and Callaghan 2019 [10] found that Irish adolescents prioritised affordability and choice when selecting food outlets, while focus groups with London students reported that proximity to fast-food outlets strongly influenced purchasing decisions, with 200 m being the maximum distance most were willing to walk [11]. Another London-based study found that low-cost was a major driver, with students often bypassing school meals in favour of cheaper ‘meal deals’ purchased from external food outlets on their way home [25].

While these international studies highlight important influences on adolescents’ food purchasing behaviours, there remains a lack of qualitative research exploring how Australian adolescents perceive and engage with their external school food environment. Addressing this gap, the present study conducted focus groups with secondary students in Perth, Western Australia, to examine their perspectives on the external school food environment and how it shaped their associated food-related behaviours.

## Methods

### Sampling and recruitment

Focus groups were conducted in June 2024 with students from an independent public co-educational secondary school (years 7–12) located in the outer suburbs of Perth, Western Australia. This school was selected due to its proximity to a retail food precinct. It should be noted that between 2018 (when the school was established) to 2024, the area surrounding the school transitioned from a largely underdeveloped area to a more densely built suburb. This included substantial residential development but also the establishment of a commercial precinct adjacent to the school. The school Principal provided written consent for the school’s participation. The school operated a ‘closed gate’ policy, whereby students (except Year 12s) were not permitted to leave the school grounds to purchase food from external outlets. This was in addition to the State Government’s mandated *Healthy Food and Drink Policy* [26], which restricts unhealthy food items across public schools in Western Australia. A detailed study information sheet and consent form were distributed to students in years 7–12 by the project’s school liaison staff member. Written parent/carer consent and written student consent were obtained prior to participation. The study was approved by Human Research Ethics Committees at the University of Western Australia (Reference no. 2020/ET000264), Edith Cowan University (Reference no. 2024-05646-TRAPP); and the WA Department of Education (Reference no. D23/1309869).

### Data collection

A semi-structured discussion guide was developed by the research team, which included experts in nutrition and qualitative research. The discussion guide addressed students’ perceptions of their external school food environment and their food-related behaviours. The focus groups included three activities to encourage active participation. Activity 1 required the students to view photographs of the external food outlets located near the school and indicate (using a sticker) whether the students or their friends visit these outlets before and/or after school. The factors influencing their decision to visit each outlet were discussed and the students were subsequently asked to rank these factors in order of influence (Activity 2). Students were then asked to collectively consider the healthiness of the outlets and rank the outlets in order of their perceived healthfulness (Activity 3), with 1 being ‘healthy’ and 10 being ‘unhealthy’. The focus groups ended with a discussion around the students’ suggested strategies to improve their external school food environment to better support students to make healthier food choices.

The focus groups were stratified by year level to facilitate free discussion and were completed on-site at the secondary school in a separate classroom in the school library. All focus groups were facilitated by three researchers; two (SM, FK) were moderators, while the other (JM) took notes and recorded non-verbal communication.

Three of the four focus groups were audio-recorded and ranged from 39 to 53 min (mean = 44 min). The fourth focus group was recorded in written format as parents of the students in this focus group did not give consent for it to be audio-recorded.

### Data analysis

Initially, content data analysis was undertaken to collate students’ answers from the three focus group activities to obtain frequencies related to food outlet use before and after school; the ranking of factors influencing students’ food outlet patronage; and the ordering of outlet healthfulness. Next, the audio recordings were transcribed verbatim by the first author (SM) and verified by a second person (SE) to ensure accuracy against the original audio recordings. The focus group transcripts were then imported into the qualitative research software, QSR NVivo, and thematic analysis was undertaken by two authors (SM, FK) guided by Braun and Clarke’s six steps of reflexive thematic analysis [27]. First, the authors became familiar with the data by reading and re-reading the transcripts. During this process, inductive coding was employed, allowing codes to be generated from the data without the use of a pre-existing coding framework. Codes were reviewed and grouped into themes. Both coders (female) brought their own experiences and assumptions to the analysis and engaged in ongoing reflexive discussion, exploring how their perspectives shaped theme development.The final themes were reviewed and mapped against pre-determined concepts, the 5As (Accessibility, Availability, Affordability, Acceptability and Attitude) framework, which has previously been applied in school food environment research [11].

## Findings

Four focus groups with 7–9 participants in each group were conducted (*n* = 31;67% females).

The external food environment comprised ten food outlets: fast-food/take-away outlets (*n* = 6), convenience store (*n* = 1), supermarket (*n* = 1), restaurant (*n* = 1), and café (*n* = 1) across two distinct areas, both located approximately 150 m from the school.

Table 1 illustrates the overarching themes identified from the focus group data, which explored students’ perceptions of their school food environment and food-related behaviours.

**Table 1:** Students’ perceptions of their school food environment and food-related behaviours: Concepts and themes

Concept	Themes
Accessibility	Proximity and public transport links Opening hours Wait time Amenities and social space
Availability	Types of food Meal deals School food and nutrition policy
Affordability	Cost of food
Advertising	Outdoor signage and advertising Social media
Acceptability and attitudes	Perceptions and odour of outlets Social influences and peer pressure Anti-social behaviour

### Accessibility

The proximity of the food outlets to the secondary school was cited as a key reason students visited an outlet: “*They’re just really close and you can walk from school*” (Student #1, Male, Year 7).

Several noted that they had not considered buying food before or after school until the fast-food outlets were established in 2021: *“Before people would just go home*…*it was kind of good*,* it saved money”* (Student #5, Female, Year 11).

Access was also facilitated by public transport links, with the school bus stopping directly outside the outlets: “*There’s like a bus that goes right behind [supermarket]. And you get off there and walk down the steps*,* and [supermarket] is right there”* (Student #1, Male, Year 7).

Some students described deliberately catching an earlier bus to allow time to visit the supermarket or fast-food outlet before school: “*[Supermarket] opens at 8 and the bus*,* the early one…that I sometimes catch*,* it comes and you wait for five or eight minutes [until the supermarket opens]”* (Student #1, Male, Year 7).

Outlets’ opening hours also shaped food purchasing behaviours, particularly before school. The 24-hour convenience store and 24-hour fast-food outlet were reported as the most visited across year levels, while students frequently waited at the supermarket until it opened in the morning: *“People wait to go into [supermarket] …they wait in line until it opens”* (Student #2, Female, Year 12).

By contrast, access during school hours was limited by the school’s ‘closed gate’ policy, with only the school canteen available for purchases. A notable exception was Year 12 students, who were permitted to leave school early on Fridays, providing opportunities to visit external outlets during the day: “*Most of the people I see [at the outlets during lunchtime] are Year 11s and 12s like on Fridays”* (Student #6, Male, Year 11).

Wait time also influenced students’ decisions to access external outlets. Students preferred venues where food and drinks were available quickly, such as the supermarket, fast-food outlets and the convenience store:

*“…the people who go to [supermarket] and [fast-food outlet] are not really going there to have a pizza or a kebab*,* they’re going for something that’s quick to have…”* (Student #2, Male, Year 10).

Slushies [a partially frozen drink made with crushed ice and syrup] from the convenience store were described as especially popular on hot days because they could be purchased quickly:

*“Yeh*,* [convenience store] is packed on hot days…people are buying slushies”* (Student #4, Male, Year 8) and “…*especially on days it’s hot you want it [slushie] nice and quick”* (Student #5, Female, Year 11 student).

Outlets with longer preparation times, such as the pizza and kebab shops, were less frequently visited, except by Year 12 students who had more time available on Fridays: *“most people will like go there to get lunch on a Friday because it’s pretty good…and we’ve got more time”* (Student #6, Male, Year 11).

Students also described accessing outlets for purposes beyond food, drawing on the amenities and social spaces they provided. For example, older students with cars used the shopping centre car park during the day:

“*There is dedicated student parking at the school*,* but since I go to [supermarket] to shop*,* for convenience after*,* I may as well park at the front”* (Student #2, Female, Year 12).

Employment at nearby fast-food outlets provided additional incentives:

*“…another reason people would want to go there is because they get the discounts. And if one person in a friend group works at [fast-food outlet]*,* they can get the discount for everyone”* (Student #6, Male, Year 11).

Outlets also functioned as social spaces and safe environments. Students described using them for shelter, air-conditioning, or as a place to meet friends:

*“There’s aircon. On cold days*,* shelter…”* (Student #1, Female, Year 12), and: *“at [fast-food outlet]… [fellow students] hang out there.…”* (Student #6, Male, Year 11).

Hanging out at the outlets was especially common among older students, sometimes because of the social appeal: “*… because it looks cool…”* (Student #3, Male, Year 11). This was consistent with findings from Activity 2, in which the Year 9/10 group and Year 11/12 group ranked ‘hang out’ as their first and second reason for visiting an outlet, respectively.

### Availability

Types of food were a salient factor in determining whether students visited outlets near the school. The convenience store was described as, “*where people go to get slushies before and after school…and energy drinks”* (Student #2, Female, Year 12). The supermarket was also popular due to its wide variety of snacks (e.g., confectionary, crisps and sugar-sweetened beverages), as one student explained: “…*this one kid*,* he brings a whole bag that goes in empty*,* and they come out it’s like full…of lollies”* (Student #2, Male, Year 10). Before school, fast-food outlets were commonly used to purchase breakfast items, such as frappes, coffee and burgers.

Meal deals were another strong influence on students’ decisions to frequent fast-food outlets. As one student observed: *“the meal deals draw people in…if there weren’t meal deals that would be less appealing”* (Student #3, Male Year 11). New menu items also created interest, with one student noting: *“You just went because it was a new thing*,* right?”* (Student #1, Female, Year 12) to which another student added *… “Yeah. We just tried one of the new items…”* (Student #8, Female, Year 12).

School food and nutrition policy also shaped student’s choices. The school prohibited students from bringing in fast-food, bulk confectionary, slushies, energy drinks and soft drinks, with these items confiscated or discarded if discovered. However, students described strategies to evade the rules: *“Students put drinks in their bag and drink them later [during lunchtime]”* (Student #4, Male, Year 8). Some even reported pouring purchased drinks into personal water bottles to avoid detection. The inconsistency of enforcement was also noted: “*the teachers tell them to put it in the bin but*,* like*,* nobody does*” (Student #1, Female, Year 12).

### Affordability

Cost of food was mentioned across all year levels as a key factor influencing decisions to visit the external food outlets. Slushies from the convenience store were considered especially cheap (AUD$1.50), snacks from the supermarkets were described as having: “*decent prices*” (Student #5, Female, Year 11) and the fast-food outlet was perceived as affordable: “*Like a dollar for a cheeseburger”* (Student #10, Male, Year 11).

In contrast, certain outlets were considered too expensive, such as the kebab shop and juice bar: “*Yeah*,* [juice bar] is so expensive for the quality*,* when you could go to like [convenience store] and get like a slushie for [AUD] $1…”* (Student #2, Female, Year 12).

The supermarket was generally regarded as offering better value compared to both the school canteen and the convenience store. Students highlighted price differences in popular items: “…*[flavoured] milk at the canteen is like $6 but you can get some for $3.5 at [supermarket]”* (Student #4, Male, Year 7). “*A large [slushie] at the canteen would be like $5 and then at [convenience store] it would be $1.50”* (Student #6, Female, Year 9).

### Advertising

Outdoor signage and advertising reinforced the visibility of the retail food precinct located near the school, particularly the fast-food outlets. Students described being able to see signage from within the school grounds:

*“My friend says every day that she wants to go to [fast-food outlet] …and it’s probably cause we can see [the outlets] from our home room”* (Student #7, Female, Year 9).

For some, this visibility triggered hunger:

“*…I’m just looking out the window and you just see them [fast-food outlet sign] … it makes you hungry…”* (Student #2, Male, Year 10).

Similar experiences were reported on the journey to school, with one student noting:

*“I walk to school*,* and I can see the [fast-food outlet] sign like halfway through my walk. It’s a k’s [1 kilometre] walk…and you feel like eating [fast-food] …because you see it for a solid 500 metres”* (Student #6, Male, Year 11).

In addition to fixed signage, students were also exposed to outdoor food advertising on buses passing the school.

Students also highlighted the constant presence of food advertising through their social media feeds. One student explained: “*It’s just ads. We get so many [fast-food] ads because of our phones listening to us talking about it.* (Student #2, Male, Year 10).

### Acceptability and attitudes

Perceptions of outlets shaped where students chose to go, with some outlets seen as more acceptable or desirable than others. For example, the fish and chip shop, chicken and chip outlet and pizza restaurant were described as places students would visit with their family for dinner, whereas the convenience store and other fast-food outlet were considered more socially desirable destinations. Students across all year levels commented that: *“you’d always find a crowd of students after school at the fast-food outlet”* (Student #5, Female, Year 11).

Some senior students also commented on the odour of nearby fast-food outlets:

*“Yeah*,* coming to school like 8:00am and smelling the fryer* …*the smell affects like how you feel…and your appetite”* (Student #2, Female, Year 12).

Another student noted that: *“Yeah*,* you can smell it [fast-food] from the bus stop”* (Student #5, Female Year 11).

Social influences and peer pressure also played a key role in shaping purchasing behaviour: “*Like if your friends are going then you’ll wanna go as well”* (Student #6, Female, Year 9).

There was also social pressure to select particular foods. Healthier options were often stigmatised, with students feeling they might be judged by peers: *“why are you getting a salad?”* (Student #4, Male, Year 8). Although students ranked the fast-food outlets and convenience store as the least healthy outlets, the healthiness of outlets was not considered an important factor in their choices: “*I don’t think any kid our age is going to say ‘I’m going to go to that healthy place instead of [fast-food outlet] because you know I want to eat healthy today’. They’re not really going to want to…”* (Student #2, Male, Year 10).

Anti-social behaviour was another sub-theme that emerged, particularly among middle high school students. Several described incidents of food being thrown within the outlets or physical fights taking place in the convenience store car park: *“A lot of our school fights actually happen there…”* (Student #2, Male, Year 10). Shoplifting was also mentioned as a reason some students visited the convenience store or the supermarket: *“since you’re able to just grab and go. It’s really easy to steal”* (Student #1, Female, Year 9). Other students speculated on the extent of the issue, with one reporting: “*yeah apparently*,* AUD$13*,*000 in one week got stolen of food*” (Student #5, Female, Year 11 student).

These behaviours had consequences, with some outlets banning school students, introducing security guards or limiting the number of students permitted inside at one time. Older students expressed disdain towards those responsible, with Year 11 and 12 students suggesting that offenders should be banned, named and even publicly shamed.

### Strategies to improve the healthfulness of the external food environment

When asked how they could be supported to make healthier choices in the external school food environment, students suggested a range of strategies:

Regulating outlet availability was seen as important, with several students proposing restrictions to prohibit and/or limit the number of unhealthy food outlets near schools, with students noting:

*“…no outlets should be allowed to be built”* (Student #3, Year 7, Female), and: “*…you shouldn’t have so many unhealthy options near a school…”* (Student #3, Year 9, Male).

Alongside restrictions, students expressed interest in the introduction of alternate outlets offering healthier options, such as Mexican-style outlets, smoothie/juice bars, and sandwich chains.

Pricing and promotions were also recognised as powerful influences. Students noted that the affordability of meal deals was a major drawcard, *“…if there weren’t meal deals*,* that would be less appealing”* (Student #3, Male, Year 11). They suggested that subsidies for healthier foods, coupled with higher prices for unhealthy items could shift purchasing behaviours. In-store promotions highlighting healthier options were also identified as a way to encourage better choices.

Finally, students emphasised the role of education and awareness campaigns. They recommended initiatives that make the health risks of frequent sugary food and drink consumption more visible, with one student reflecting on the consequences of habitual intake, *“…constantly starting your day like that*,* and eating those kinds of things and pumping up on sugar*,* sugary snacks…”* (Student #2, Female, Year 12).

## Discussion

This qualitative study of Western Australian secondary school students’ explored perceptions of the external school food environment. Students described these environments as highly accessible, affordable and socially desirable, yet dominated by unhealthy food and beverage options and advertising. Their accounts revealed that convenience, pricing, peer influence and marketing cues strongly shaped purchasing decisions. These findings suggest that that outlets near schools are not benign and may contribute to obesogenic environments that influence adolescents’ food choices during a critical stage of life. This extends international evidence by providing new insights into how Australian adolescents perceive and navigate these environments, highlighting dimensions that are difficult to capture in quantitative studies.

Our findings also demonstrate the powerful effect of environmental change. Students reported that before the establishment of the nearby food outlets, they had not considered purchasing food before and after school. Since the outlets opened, however, visiting them had become a routine and socially normalised behaviour. This shift illustrates how changes in the local food environment may shape, rather than merely reflect adolescent behaviours, as perceived by students. These findings highlight the role of urban planning approaches, informed by students’ calls to limit the number of unhealthy food outlets near schools, in shaping healthier external school food environments and reducing the normalisation of unhealthy purchasing behaviours during adolescence. This is particularly concerning because adolescence is a life stage where dietary habits track into adulthood [7]. Exposure to environments saturated with cheap, unhealthy and highly marketed options, may encourage patterns of consumption that begin before school and extend into the afternoon. Overall, our findings are consistent with international research showing that proximity, affordability and promotions are key drivers of adolescent food purchasing [10, 11]. The type and price of food available at outlets strongly influenced students’ choices. Convenience stores were popular for cheap slushies (AUD$1.50) and energy drinks, and supermarkets were valued for their wide variety of low-cost snacks, confectionary and sugar-sweetened beverages. Fast-food outlets attracted students through meal deals, new menu items and low-priced specials, such as AUD$1 cheeseburger. These findings are consistent with insights from focus groups with adolescent students in Ireland, who were similarly drawn to outlets offering meal deals and price discounts [10]. Students consistently compared prices across outlets and with the school canteen, perceiving supermarkets as better value while avoiding outlets like the kebab shop and juice bar due to their higher cost. This price sensitivity reflects the ‘consumer mentality’ of adolescents [11, p.215] and their growing financial independence and autonomy [20, 21]. Adolescents’ emphasis on affordability demonstrates the importance of fiscal levers such as taxes on sugary drinks or subsidies for healthy options which international evidence shows can shift consumption patterns [33].

Students’ preference for, and access to, external food outlets was strongly shaped by opening hours and school policies. Outlets that opened early were particularly attractive before school, while the school’s ‘closed gate’ policy limited access during the school day. Quick-service options were especially favoured, reflecting students’ need to maximise limited time before and after school. Similar evidence from Ireland and the UK [10, 11] shows that adolescents are more likely to purchase food from outlets that fit within these narrow time windows. In addition to meeting practical needs, these food outlets often served as important social spaces, providing students with a “third place” to socialise and spend time with peers outside of home and school, fostering social connection, and a sense of community, which are particularly important in adolescence [28]. The location of bus stops and timing of bus schedules also facilitated students’ access to the food outlets, highlighting the role of urban planning and transport infrastructure in shaping food acquisition behaviours among youth.

This study revealed that food outlets near schools are not just places where adolescents buy food, they have become unsupervised social hubs where risky behaviours are normalised. While older students used these outlets to socialise and assert independence [29], they also reported theft, vandalism and even physical fights. These behaviours are rarely considered in food environment research, yet they highlight the problematic role these spaces can play in shaping adolescent development. Rather than supporting healthy social interaction, our findings suggest that clusters of outlets appear to foster both poor dietary habits and anti-social behaviour. The placement of such precincts directly opposite schools may not be a neutral planning decision, as it can create environments that potentially undermine young people’s health and wellbeing. Another novel finding was the sensory dimension of the external school food environment. Students commented on the odour of the nearby food outlets. While rarely explored in school food environment research, this finding is noteworthy given evidence that smell is closely linked to food consumption [30] and that food odours can independently stimulate appetite [31]. In addition, students noted the visibility of outdoor advertising, with large fast-food signs seen from classroom windows, on buses and along bus routes acting as constant cues to purchase food and visit outlets. Taken together, these findings suggest that both sensory and visual exposures reinforce unhealthy food purchasing and highlight the need to consider odour and advertising as key features of the obesogenic environment surrounding schools.

### Policy and practice recommendations

The perspectives offered by students point to several avenues for improving the healthfulness of external school food environment. Students supported restrictions on unhealthy food outlets near schools, echoing local and international calls for school exclusion zones and ‘takeaway management areas’ [32]. Evidence from the UK demonstrates that such planning controls can be effective. For example, Gateshead Council’s policy introduced exclusion zones around schools and restrictions based on retail density, which led to a reduction in the proportion and density of fast-food outlets [33] and was associated with lower childhood obesity prevalence in areas with a high concentration of outlets [34]. At present, no comparable measures exist in Australia, despite increasing recognition of the role of urban planning in shaping population diets. The findings from this study contribute adolescent-driven evidence that may inform future government action in this area.

Students recognised the influence of pricing, suggesting that unhealthy food should be made less affordable, and healthier food, subsidised. These insights are supported by international evidence; systematic reviews of pricing strategies confirm that taxes, price manipulations, and subsidies can significantly alter purchasing behaviour [35]. The fact that adolescents themselves identified cost as both a barrier and lever for change highlights their awareness of the commercial determinants of health and strengthens the case for fiscal policies targeting unhealthy food and drinks.

Advertising was another area of concern, particularly the pervasiveness of outdoor signage visible from classroom windows and bus routes [36]. The World Health Organization have called for comprehensive restrictions on food marketing to children, citing overwhelming evidence of its harmful effects [37]. The ubiquity of signage and digital marketing described by students shows the limitations of voluntary industry codes [38] and reinforces the need for stronger regulatory measures that are consistent with WHO’s call for comprehensive restrictions on unhealthy food marketing to children. Students in this study recommended restrictions on in-store promotions, including point of sale (‘checkouts’) and end-of-aisle displays, which is noteworthy given that research shows around 80% of supermarket display space in Australia promotes unhealthy foods [39], and that placement and promotion strategies significantly influence purchases [40]. Although early UK efforts to regulate in-store promotions have faced challenges [41], these measures form an essential component of a comprehensive policy response [37].

### Strengths and limitations

A key strength of this study is that it amplifies the voices of adolescents, an often-overlooked group in food environment research through direct qualitative inquiry. The purposive selection of a school adjacent to a retail food precinct offered a unique opportunity to examine how adolescents interact with outlets on a daily basis. The use of focus groups enabled peer-to-peer discussion, which is well suited to capturing adolescent perspectives [42]. To limit the possibility of students providing socially desirable responses (e.g., reluctance to admit to stealing, eating unhealthy food), teachers were not present during the focus group discussions and students were assured of their anonymity and confidentiality.

However, limitations should be acknowledged. First, the sample was skewed towards females. Second, while focus groups encouraged rich discussions, some students may have been reluctant to express unpopular views in front of peers, despite efforts to stratify groups by age and year group and assure confidentiality. Third, focus groups were stratified by year level and the analysis prioritised identifying common themes across groups rather than conducting age-specific comparisons. As students’ autonomy and interactions with the external food environment are likely to vary by age, some age-related nuances may have not been fully captured. Finally, as the study focused on a single school in Perth and may have limited transferability to other geographic or socioeconomic contexts, further research is needed to explore if themes are applicable to other school settings.

## Conclusion

This study provided new insights into how adolescents perceive and navigate the external school food environment in Australia. Students described these environments as cheap, convenient and socially compelling, yet overwhelmingly unhealthy. Their accounts highlight how outlet proximity, pricing, promotions and marketing cues converge to normalise poor dietary habits and even risky behaviours such as theft and fighting.

Crucially, students articulated clear strategies for change, including restricting unhealthy food outlets near school, subsidising healthier foods and regulating advertising and promotions. These suggestions align with international best practice and highlight that young people themselves recognise the structural drivers of their dietary choices.

Left unchecked, external school environments will continue to reinforce poor diet and harmful social norms. Urgent, coordinated policy action is required. Restricting unhealthy outlets near schools, reshaping pricing and promotions and amplifying healthier alternatives are not optional – they are essential if we are to protect adolescents from a food system designed to exploit them.

## Data Availability

The datasets used and/or analysed during the current study are available from the corresponding author on reasonable request.
